# A Spatio-Temporal Graph Convolutional Network Model for Internet of Medical Things (IoMT)

**DOI:** 10.3390/s22218438

**Published:** 2022-11-02

**Authors:** Dipon Kumar Ghosh, Amitabha Chakrabarty, Hyeonjoon Moon, M. Jalil Piran

**Affiliations:** 1 Department of Computer Science and Engineering, Brac University, Dhaka 1212, Bangladesh; 2 Depatment of Computer Science and Engineering, Sejong University, Seoul 05006, Korea

**Keywords:** Internet of Medical Things (IoMT), healthcare, human action recognition (HAR), graph convolutional network (GCN)

## Abstract

In order to provide intelligent and efficient healthcare services in the Internet of Medical Things (IoMT), human action recognition (HAR) can play a crucial role. As a result of their stringent requirements, such as high computational complexity and memory efficiency, classical HAR techniques are not applicable to modern and intelligent healthcare services, e.g., IoMT. To address these issues, we present in this paper a novel HAR technique for healthcare services in IoMT. This model, referred to as the spatio-temporal graph convolutional network (STGCN), primarily aims at skeleton-based human–machine interfaces. By independently extracting spatial and temporal features, STGCN significantly reduces information loss. Spatio-temporal information is extracted independently of the exact spatial and temporal point, ensuring the extraction of useful features for HAR. Using only joint data and fewer parameters, we demonstrate that our proposed STGCN achieved 92.2% accuracy on the skeleton dataset. Unlike multi-channel methods, which use a combination of joint and bone data and have a large number of parameters, multi-channel methods use both joint and bone data. As a result, STGCN offers a good balance between accuracy, memory consumption, and processing time, making it suitable for detecting medical conditions.

## 1. Introduction

With the emergence of the Internet of Medical Things (IoMT), the continuous monitoring of patients has become increasingly accessible in everyday life [1,2,3]. IoMT enables the integration of IoT communication protocols with medical equipments and systems, enabling remote, real-time, and intelligent patient monitoring and treatment [4,5]. Physicians are able to treat more patients with real-time patient monitoring, and patients are reassured that someone is always watching out for them. A rapid improvement in wearable technologies has helped to develop intelligent and real-time healthcare services, including Parkinson’s disease monitoring, Alzheimer’s disease monitoring, and fall detection [6,7,8,9]. It is possible to immediately and accurately detect physiological states with wearable technologies, but some acute and dormant illnesses, such as lumbago and neuralgia, remain indefinable or prohibitively expensive to treat [10].

Computer vision (CV) methods are capable of excavating these symptoms for standard medical measures if comfort and functionality are taken into account [11]. Real-time patient monitoring systems can use human action recognition (HAR) as a context-aware application. With HAR in smart healthcare environments, action recognition will be easier from visual data as well as sensor data, such as Microsoft skeleton data. It is necessary to deploy HAR models on GPU-enabled edge devices, such as Jetson Nano, Jetson TX2, and Jetson AGX Xavier.

Most hospitals, clinics, and healthcare centers today have video cameras that can be used to monitor patients. Monitoring patients and manually detecting their conditions in real time is time-consuming and expensive, and informing the appropriate authority in case of an emergency is time-consuming and expensive. Moreover, in the event of an emergency, informing the appropriate authority may take some time. On the other hand, an automated action recognition system can do so almost immediately in an intelligent healthcare environment.

**Figure 1:**
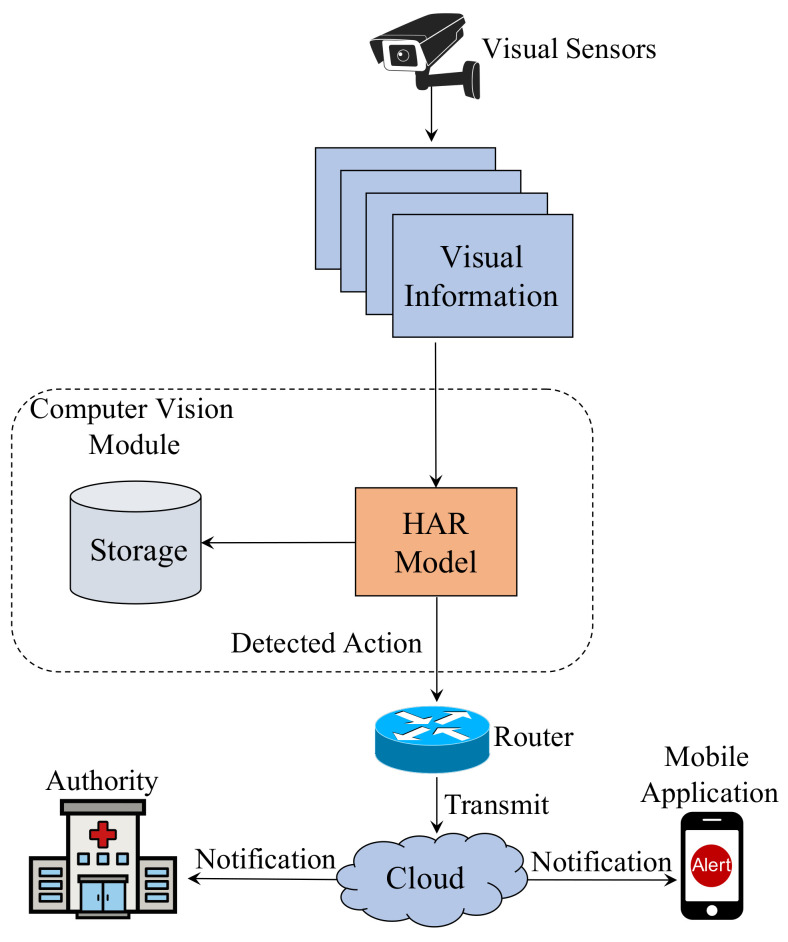
A smart healthcare system for real-time patient monitoring.

An intelligent patient monitoring ecosystem is illustrated in Figure 1. For example, Microsoft Kinect can be used to collect skeleton points from a visual sensor. Once the information is passed to the CV module, an HAR model predicts an action. Using sensor data, the CV module can recognize actions in real time. A router connects the whole system to the cloud so that it can notify the authorities in case of an emergency. By doing so, it will be possible to implement a vision-based real-time monitoring system for patients. Notifications can also be sent to a mobile application in a home surveillance system. In addition to storing predicted footage, the CV module also includes a storage system.

The skeleton data consist of 3D points from motion cameras or pose estimation technology that can be used to analyze human behavior. Since skeleton data contain fewer dimensions, it is computationally more efficient than traditional RGB videos for representing human dynamics. Furthermore, it is resilient to illumination issues, flickering clips, motion blur, and complex backgrounds [12]. We present our method for skeleton-based action recognition, which can be used in smart healthcare systems to monitor patients.

In order to solve this problem, different deep learning (DL)-based approaches have been proposed. Skeleton points are traditionally represented by joint-coordinate vectors and passed to recurrent neural networks (RNNs) [13,14] or pseudo-images from skeleton data are passed to convolutional neural networks (CNNs) [15,16]. If skeleton points are represented as graph structures, then their full potential can be exploited. Graph convolutional networks (GCNs), which perform convolutional operations on graphs, have also gained considerable attention [17,18].

In recent years, GCNs have been successfully applied to skeleton-based recognition tasks with success [19,20]. The existing methods, however, are computationally inefficient and suffer from a slow execution speed and large model sizes. Some methods combine multiple input streams, such as bone and joint data, which make the models even heavier and restricts their application to real-life applications, including patient monitoring. Modern GCNs construct spatial graphs from skeleton data and pass them into spatial GCNs. In order to obtain temporal features, they are passed to a temporal convolutional layer. It is possible that spatial GCN does not extract any significant temporal features during sequential feature extraction. There is therefore a loss of information because spatial and temporal features are not extracted from the same spatio-temporal feature state.

**Figure 2:**
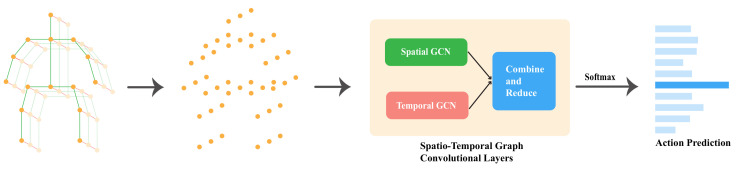
The end-to-end pipeline of STGCN.

This paper introduces a novel architecture called a redefined spatio-temporal graph convolutional network (STGCN) for skeleton-based HAR, which independently extracts relevant spatial and temporal information, merges them, and detects action. We propose a spatial and temporal adaptive graph convolution operation [20] that extracts significant spatial and temporal features independently from skeleton joint data, as illustrated in Figure 2. In the proposed model, spatial, and temporal adaptive graph convolutional layers are combined to extract significant spatial and temporal features from the same spatio-temporal position. A further benefit of our model is that it uses only one stream of input, as opposed to other multi-channel methods, which use multiple input streams. As compared to other multi-channel methods, our method ensures better feature extraction since the same type of layer is applied multiple times to multiple input streams and then combined later.

In order to demonstrate the effectiveness of our proposed model, we performed extensive experiments on a skeleton-based action recognition dataset, namely the NTU-RGBD [13] dataset. Based on the NTU-RGB dataset, our model achieves state-of-the-art results. Furthermore, we demonstrate the applicability of our model in a real-world environment by measuring its performance on edge devices, such as the Nvidia Jetson Nano. Due to its low computational complexity, reduced parameter size, and fast processing speed, our model is ideal for dynamic detection and deployment in the real-time monitoring of patients in intelligent healthcare ecosystems.

Our main contributions are summarized as follows.

- In the context of IoMT, an efficient spatial and temporal feature extraction framework for HAR is introduced, together with a framework for utilizing the features.
- A novel architecture, STGCN, is proposed to enable the independent extraction of spatial and temporal features. Due to its reduced number of parameters and efficient feature extraction method, our model extracts spatial and temporal features from only joint-level information.
- Finally, we provide a strong framework for skeleton-based HAR. We demonstrate with extensive experimentation and analysis that our models achieve competitive accuracy with state-of-the-art models. The baselines we established should be useful to future research on skeleton-based HAR and vision-based patient monitoring.

The rest of the paper is organized as follows. Section 2 represents an overview of the related work. Section 3 explains the detailed architecture of STGCN. Details of our experimental setup are described in Section 4. Then, we show the results and analysis of our experiments in Section 5. Finally, we our conclusion is provided in Section 6.

## 2. Related Work

For HAR and skeleton-based HAR, two types of methods can be used, including those that use handcrafted features and those that use deep learning. We briefly review both categories of methods in this section. For healthcare systems, we also study vision-based methods.

### 2.1. Human Action Recognition

#### 2.1.1. Hand-Crafted Feature-Based Methods

In the past, HAR methods have relied on manually extracting features from motion sequences. Motion energy image (MEI) and motion history image (MHI) are two new methods for action representation introduced in [21]. As an extension of HOG features in the spatial and temporal dimensions, the 3D histograms of gradients (3DHOG) were proposed to represent human action [22]. In addition to hand-crafted feature-based models, spatial-temporal interest points (STIPs) were used to represent human actions based on their similarity to clips in space–time dimensions [23].

#### 2.1.2. DL-Based Methods

In recent years, DL-based methods gained a lot of attention due to their improved accuracy and performance. There was widespread use of CNNs and long short-term memory networks (LSTMs) for video understanding [24]. Two types of input are passed through convolutional layers in a two-stream CNN [25] and merged at the end for classification. A stream of the network process optical flow extracts temporal information from images. Another stream extracts the spatial information from an image.

### 2.2. Skeleton-Based Action Recognition Methods

The performances of DL-based methods such as RNNs, CNNs, and GCNs are remarkably better than those of approaches based on handcrafted features [26].

#### 2.2.1. RNN-Based Methods

Sequential data can be modeled using RNNs such as LSTMs and gated recurrent units (GRUs). A sequence of vectors was used to model skeleton data for skeleton-based action recognition [27,28]. In [29], Hong et al. proposed a two-stream RNN architecture to model the skeleton data’s temporal dynamics and spatial configurations.

#### 2.2.2. CNN-Based Methods

Generally, CNNs use structured data in the form of 2D or 3D models. Thus, skeleton data have been manually transformed into pseudo-images and passed into CNN-based models [30,31]. Convolution operations in CNN-based models, however, were limited to neighboring joints, so correlations with joints other than neighbors could not be represented due to the representational constraint.

#### 2.2.3. GCN-Based Methods

GCN performs convolution operations on graphs and has recently attracted a lot of interest [17,32]. Since skeleton data can be easily represented as graphs, GCN-based methods gained popularity in skeleton-based action recognition. A spatial temporal GCN (ST-GCN) model was proposed by Sijie et al. in [19], which constructs a spatio-temporal graph in which joints are vertices and edges are connected with natural connections in human body structures and time. The two-stream adaptive GCN (2s-AGCN) uses adaptive graph convolution operations on both joint and bone data to recognize actions, as described by Lei et al. in [20].

### 2.3. Vision-Based Methods for Healthcare Services

CV is being used to develop smart and intelligent healthcare monitoring systems for patients and the elderly. To extract the spatio-temporal characteristics of human action, the authors in [33] used the Minkowski and cosine distances between joints. Their method was applied to the development of elderly monitoring systems. An architecture for medical condition detection based on skeleton data was proposed by Yin et al. in [10]. For detecting such actions, they proposed an optimized view of an adaptive LSTM network with additional subnetworks. Vision-based patient monitoring systems have also been developed using CNNs [34]. Furthermore, Gao et al. in [35] developed a method to detect medical conditions by combining 3D CNNs and LSTMs.

## 3. Proposed STGCN

Figure 2 illustrates the overall pipeline of our proposed model. The first step is to collect skeleton points from depth cameras or RGB videos using pose estimation modules. Using those points, a spatial graph is constructed, passed through spatiotemporal graph convolutional blocks, and an action is predicted.

### 3.1. Skeleton Graph Construction

A skeleton consists of a sequence of vectors representing the 2D or 3D coordinates of human joints. Using ST-GCN* [19], we formed a spatial temporal graph to represent structured information in skeleton sequences. We defined an undirected graph G=(V,E) with a skeleton sequence that consists of *N* joints and *T* frames.

**Figure 3:**
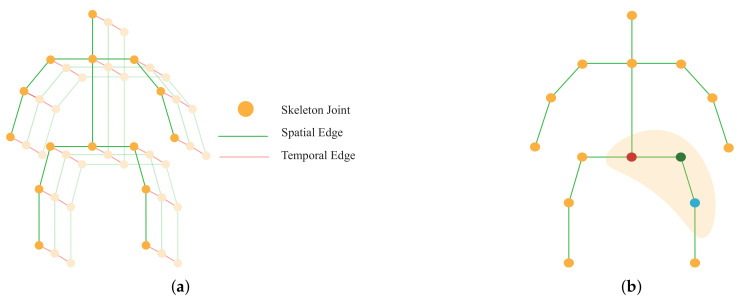
(**a**) Spatio-temporal graph of skeleton joints; (**b**) Mapping of different joints in the graph depending on their position.

In the graph, the vertices $V=\{v_{ti}|t=1,\ldots ,T,i=1,\ldots ,N\}$ consist of all the joints in a skeleton sequence. Figure 3a illustrates construction graphs from skeleton data. There are two sets of edges in the graph. The first one is called spatial edges (green lines in Figure 3a), which consists of all natural connections in the human body within a specific frame, $E_{S}=\left\{v_{ti}v_{tj}|i,j\in H\right\}$, where *H* is the set of naturally connected human joints. The other is temporal edges (red lines in Figure 3a), which are formed by connecting analogous joints between two adjacent frames, $E_{T}=\left\{v_{it}v_{i(t+1)}\right\}$. The edges in $E_{T}$ express dynamics for a specific joint *i* across *T* frames.

### 3.2. Graph Convolution

By performing graph convolution, inputs are passed through the layers of GCN to obtain high-level features. According to [19], the graph convolution operation on vertex $v_{i}$ can be defined as

(1) $$f_{out}\left(v_{i}\right)=\sum_{v_{j}\in B_{i}}\frac{1}{Z_{ij}}f_{in}\left(v_{j}\right)\cdot w(l_{i}\left(v_{j}\right)),$$

where f represents feature maps, so $f_{in}$ and $f_{out}$ represent the input and out feature maps, respectively. *v* is the vertex of the graph and *w* denotes the weighting function which is analogous to the original convolution operation. $B_{i}$ represents the set of unit distance neighboring vertices ($v_{j}$) of the corresponding vertex $v_{i}$ which take part in convolution operation with $v_{i}$. $l_{i}$ was put in ST-GCN [19] to map the variable number of neighboring vertices in $B_{i}$ to form three clusters, including the vertex itself, $C_{i}1$ (the red circle in Figure 3b), neighboring vertices closer to the center of gravity, $C_{i}2$ (the green circle), and vertices far away from the gravity, $C_{i}3$ (the blue circle). $Z_{ij}$ exists to balance the contribution of each cluster which represents the number of $C_{ik}$ in $v_{j}$.

### 3.3. Implementation of GCN

It is required that (1) is converted into the form of tensors in order to implement the GCN. The shape of skeleton features for the model is C×T×N, where *C* denotes the number of channels, *T* represents the number of frames and *N* denotes the number of vertices. To implement the GCN, (1) is transformed into the following.

(2) $$f_{out}=\sum_{k}^{K_{v}}W_{k}\left(f_{in}A_{k}\right)⊙M_{k},$$

where $k_{v}$ is the spatial kernel size, and following the above strategy, it is set to three. The matrix $A_{k}$ is defined as $A_{k}=\Lambda_{k}^{-\frac{1}{2}}{\bar{A}}_{k}\Lambda_{k}^{-\frac{1}{2}}$. ${\bar{A}}_{k}$ is the adjacency matrix for the graph of shape N×N, which contains element, ${\bar{A}}_{k}^{ij}$, indicating whether the vertex $v_{j}$ is in the cluster $C_{ik}$ of vertex $v_{i}$. $\Lambda_{k}$ is the normalized diagonal matrix, and each element of $\Lambda_{k}$ is defined as $\Lambda_{k}^{ii}=\sum_{j}\left({\bar{A}}_{k}^{ij}\right)+\alpha$. The value of α is set to 0.001 to prevent empty rows. $W_{k}$ represents the weighting function in (2) and is defined as the weight vector of shape $C_{in}\times C_{in}\times 1\times 1$ of 1×1 convolution operation. $M_{k}$ represents the significance of each vertex and is defined as the N×N attention map. ⊙ indicates dot product operation.

However, the implementation of GCN from (2) is based on predefined graph construction, which does not guarantee the optimal solution [20]. Thus, here we modify (2) according to [20] as follows.

(3) $$f_{out}=\sum_{k}^{K_{v}}W_{k}f_{in}\left(A_{k}+B_{k}+C_{k}\right),$$

where the adjacency matrix is divided into three parts:

1. $A_{k}$: this denotes the physical structure of the human body and is the same as the normalized N×N matrix $A_{k}$ in (2). In the skeletal graph, human joints are treated as vertices and they are connected according to the human body structure. The adjacency matrix, $A_{k}$, is computed to represent the skeletal graph, which determines whether there is a connection between two vertices.

2. $B_{k}$: It is also an adjacency matrix of shape N×N and the values of $B_{k}$ are parameterized and they learn throughout the training process along with other parameters. Although $B_{k}$ can play the similar role of $M_{k}$ in (2), it is more flexible and efficient than $M_{k}$. The model learns to fully focus on the recognition task and target individual information to form different layers with the help of this adjacency matrix. The initial value of $B_{k}$ is set to 0 and during the training process, $B_{k}$ learns the parameters depending on a specific action class. This helps $B_{k}$ to learn to detect the most significant joints for a particular action. Thus, along with the existence of a connection between joints, $B_{k}$ also learns to identify the strength of a connection.

3. $C_{k}$: $C_{k}$ learns a different graph for each sample input, and determines whether there is any connection between the two joints and the strength of the connection. It does so by calculating the similarity between the two vertices by applying the normalized embedded Gaussian function.

(4) $$f\left(v_{i},v_{j}\right)=\frac{e^{{\theta (v_{i})}^{T}\phi \left(v_{j}\right)}}{\sum_{j=1}^{N}e^{{\theta (v_{i})}^{T}\phi \left(v_{j}\right)}},$$

where *N* is the total number of vertices.

The dot product is used to find the similarity between two joints in the embedding space. In detail, first input $f_{in}$ is embedded from shape $C_{in}\times T\times N$ to shape $C_{em}\times T\times N$ with two embedding functions, θ and ϕ. Following [20], we use a single 1×1 convolutional layer as the embedding functions. The output features of these functions are reorganized and reshaped into $N\times C_{em}T$ and $C_{em}T\times N$ matrices. Then, the features maps are multiplied together to form the N×N shape matrix $C_{k}$, whose element $C_{k}^{ij}$ denotes how similar the vertex $v_{i}$ is to the vertex $v_{j}$. Following that, the values are normalized in the range of 0–1, and a softmax function is used. The whole process can be represented by the following equation.

(5) $$C_{k}=softmax\left(f_{in}^{T}W_{\theta k}^{T}W_{\phi k}f_{in}\right),$$

where $W_{\theta }$ and $W_{\phi }$ denotes the parameters of the two embedding functions, θ and ϕ, respectively.

**Figure 4:**
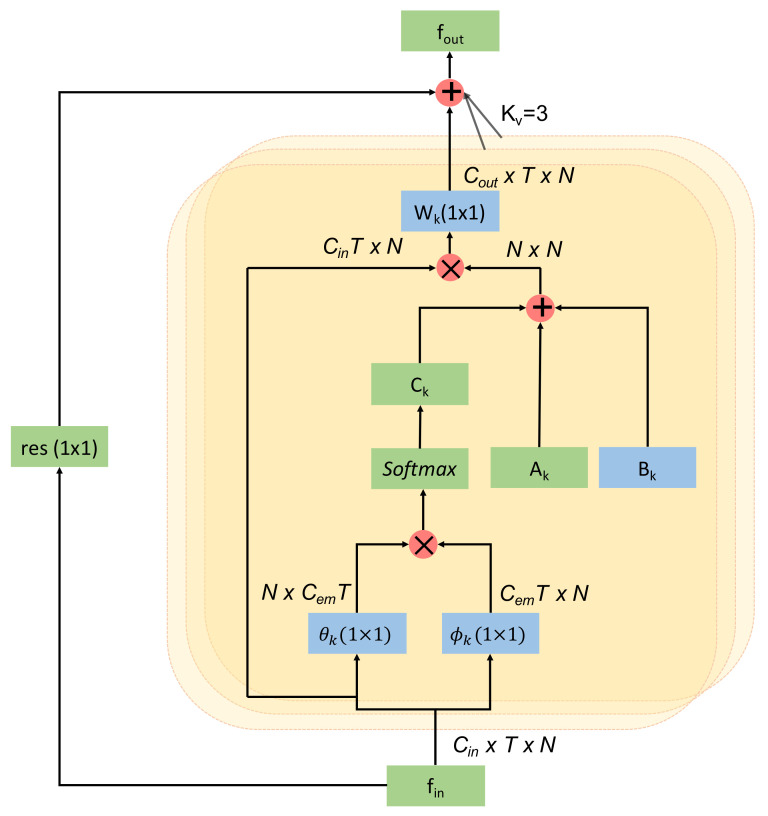
Architecture of an adaptive graph convolutional layer.

The overall architecture of the adaptive graph convolutional layer is depicted in Figure 4. The kernel size for the convolution operation ($k_{v}$) is set to three, except for $A_{k}$, $B_{k}$, and $C_{k}$, as discussed above. $W_{k}$ is the weight function introduced in (1). First, the input is transformed into an embedding space using the function θ and ϕ following (4). The output of the embedding functions are multiplied together element-wise (displayed by ⊗) to form the matrix $C_{k}$. Then, the three adjacency matrix $A_{k}$, $B_{k}$, and $C_{k}$ are added elementwise, which is shown by ⊕. The added result is then multiplied with the input and passed through an convolutional layer. Finally, a residual connection is used to insert the input feature, which improves accuracy. If the number of channels in the input and output of this adaptive graph convolution layer does not match, then a 1×1 convolution is used in the residual path to match the output channel dimension to the input channel dimension.

### 3.4. Spatio-Temporal Graph Convolutional Block

**Figure 5:**
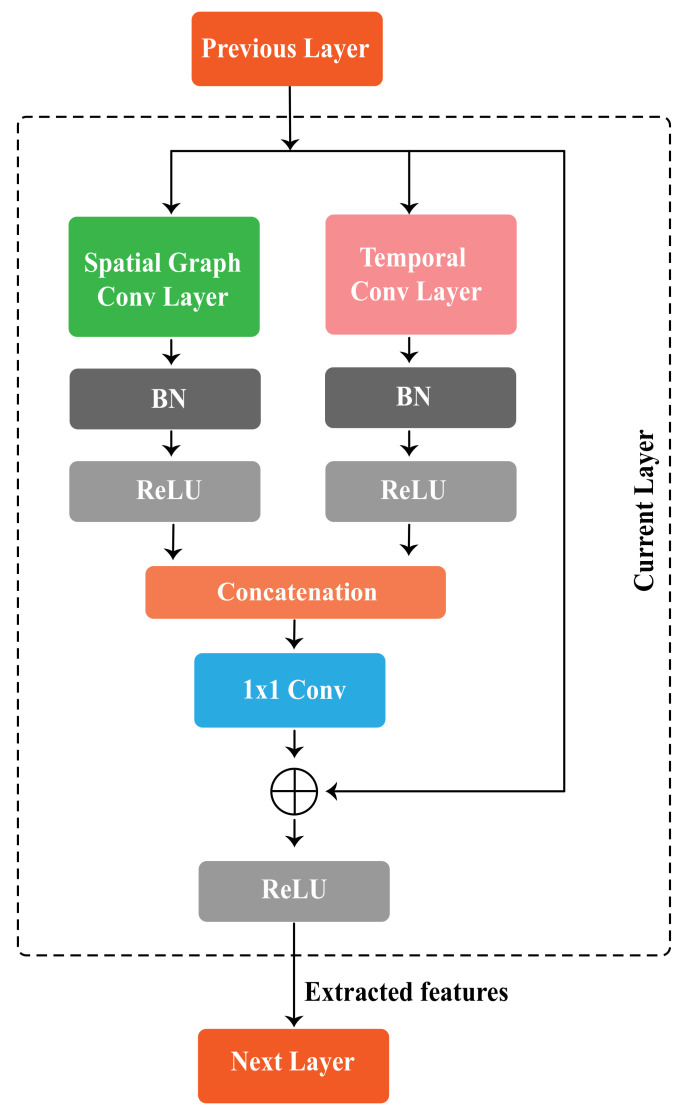
A spatio-temporal graph convolutional block of STGCN.

Each spatio-temporal graph convolutional block consists of a spatial convolution layer and temporal convolution layer. Spatial features extracted by spatial graph convolutional layers, which are implemented from (3), while temporal features are extracted by following the convolution operations for the temporal dimension from ST-GCN [19], following (2). The temporal convolution layer consists of a 2D convolution layer with $k_{t}\times 1$ kernel size, which takes the features of shape C×T×N as input. The spatial graph convolution layer performs graph convolution operation on spatial edges to extract spatial features, whereas the temporal graph convolution layer performs graph convolution on temporal edges. Figure 5 illustrates a single block of STGCN, which includes a spatial graph convolutional layer and a temporal convolutional layer. BN and ReLU layers are added to the temporal convolution layers as well as spatial convolution layers. In our proposed method, spatial and temporal features are extracted in parallel and independently from each other by our redefined spatio-temporal graph convolutional block, whereas in other methods such as ST-GCN and 2s-AGCN, the features are extracted sequentially. There is a 1×1 convolutional layer to reduce the output channel, which comes from concatenating the features extracted by spatial and temporal graph convolutional modules. Finally, to improve the performance and network stability, a residual connection is added to the block.

### 3.5. Spatio-Temporal Graph Convolutional Layers

**Figure 6:**
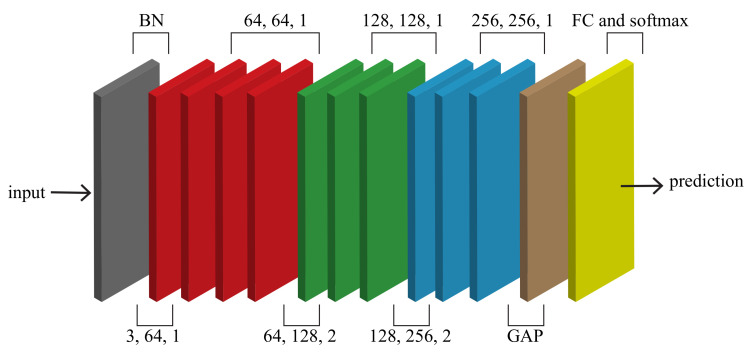
The architecture of STGCN.

As shown in Figure 6, these blocks form the STGCN network. The network consists of ten blocks. The first four blocks have 64 output channels, blocks 5–7 have 128 output channels, and the remaining blocks have 256 output channels. In order to normalize the input, we added a BN layer at the beginning. For the final prediction, a fully connected (FC) layer with a softmax function is used to combine and reduce the extracted features.

## 4. Experimental Setup

This section analyzes the performance of our model on public benchmark datasets for skeleton-based action recognition. Despite its low computational complexity and smaller memory footprint, our model outperforms the baseline models in the corresponding category.

### 4.1. Datasets

#### NTU-RGBD Dataset

The performance and efficiency of STGCN are tested on a large-scale skeleton-based action recognition dataset, NTU-RGBD [13]. RGBD consists of 56,000 action clips categorized into 60 action classes, making it the most widely used dataset for action recognition. Three cameras film each action at the same height, but at different horizontal angles: −45${}^{\circ }$, 0${}^{\circ }$, 45${}^{\circ }$. We report top-1 accuracy in two validation subsets, as suggested by the original literature [13]. First, there is the cross-subject subset (X-sub), wherein the training set and validation set are divided based on actors, including a total of 40,320 training samples and 16,560 validation samples. Then, the cross-view subset (X-view) divides the two sets according to the camera—including 37,920 training samples from the second and third cameras, and 18,960 validation samples from the first camera.

### 4.2. Training Details

The DL framework PyTorch was used to implement our model, which is a very popular and widely used framework. We trained STGCN with the stochastic gradient descent (SGD) optimizer and a Nesterov momentum of 0.9 and weight decay of $1\times 10^{-4}$. At epochs 30 and 40, the learning rate was reduced by a factor of 10. With a batch size of 16, training was completed after 50 epochs. For gradient backpropagation, we selected cross-entropy as the loss function.

There is a maximum of two people in each sample of the NTU-RGBD dataset. Whenever a sample has fewer than two individuals, the second individual is padded with zero. A sample of the dataset contains a maximum of 300 frames. Any sample that contains fewer than 300 frames is replicated and added to the sample to make the frame count 300. We train our models from scratch using the corresponding datasets.

## 5. Results and Discussion

The purpose of this section is to evaluate our model by performing extensive studies with different input types, comparing it with other standard models, and illustrating its effectiveness. Additionally, we demonstrate the efficacy of our model in an intelligent healthcare system.

### 5.1. Visualization of Feature Selection

**Figure 7:**
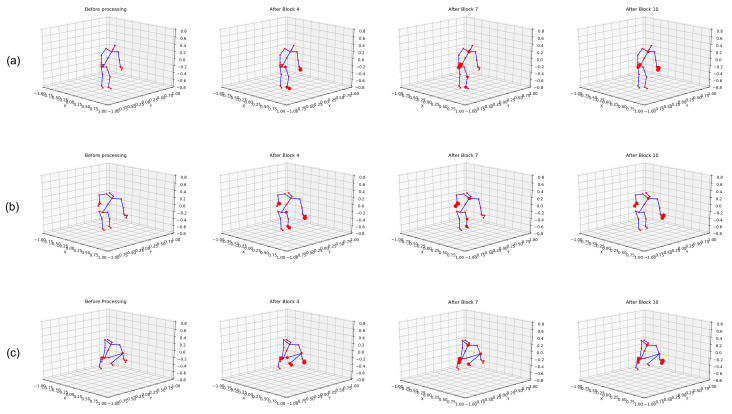
Visualization of feature extraction by STGCN. (**a**–**c**) represents the features extracted at different frames.

Our model performs feature extraction on temporal and spatial dimensions independently and combines them. Figure 7 illustrates the joints selected by our model for the action pickup. At different stages of a network, we show skeletons performing the action pickup. From each dimension, we select the joint with the highest score and count the number of selected joints. Red circles indicate the top five selected joints in the visualization. Circle size represents the number of times a joint is selected, meaning that the largest joint is the most frequently used joint.

We show three frames in Figure 7a–c for the pickup task. The extracted features highlight hand and leg joints while the body was moving downwards, and later when the body had already moved downwards, both hands were selected, indicating the pickup.

**Figure 8:**
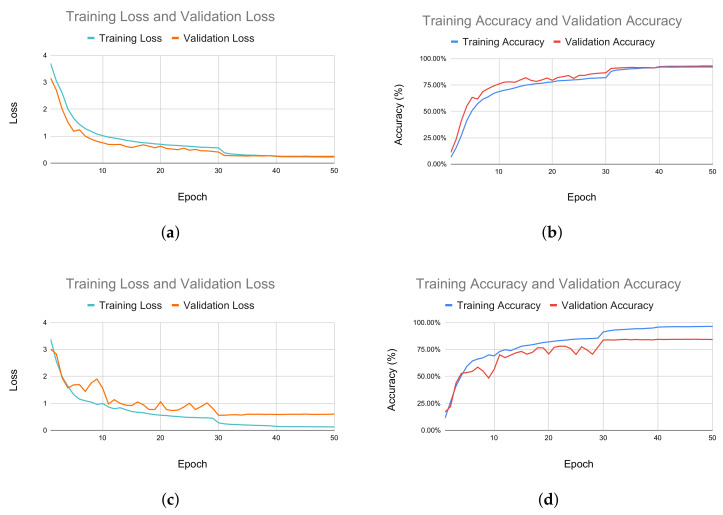
Performance measurement of the training and validation process. (**a**) Comparison between training loss and validation loss for the X-view subset. (**b**) Comparison between training and validation accuracy for X-view subset. (**c**) Comparison between training and validation loss for X-sub subset. (**d**) Comparison between training accuracy and validation accuracy for X-sub subset.

Additionally, in Figure 8, we report a comparison between the loss and accuracy of the model in the training and validation phases for both the X-sub and X-view subsets. In the X-view subset, as illustrated in Figure 8a,b, the model was initially overfitted during training, but was fixed as the training progressed. In the X-view subset, the training accuracy and validation accuracy were steady during the whole process. The same trend was noticed with loss too, as shown in Figure 8c.

### 5.2. Ablation Study

As discussed in Section 3.3, there are three types of graphs in the adaptive graph convolutional block, including A, B, and C. We perform an ablation study of our model to identify the importance of each adjacency matrix.Table 1 shows the importance of the adaptive learning of graph for action recognition and the performance of the model is hampered when any one of the three graphs is removed. Accuracy decreases most when matrix $B_{k}$ is removed. This happens because $B_{k}$ has learnable parameters, which learns to detect the most significant joint for an action. When $A_{k}$ is missing, the accuracy drops but not as much as when $B_{k}$ is removed. It happens because $A_{k}$ is predefined and $B_{k}$ learns the structure of the graph as the training progresses. The model achieves its best accuracy with all three graphs.

### 5.3. Performance Analysis with Different Input Features

We performed a study to determine which input features provide the best result for our model. As shown in Table 2, we obtain 83.8% X-sub accuracy and 91.4% X-view accuracy using skeleton bone data. Both accuracies increased when skeleton joint data was used. We obtained 84.5% X-sub accuracy and 92.2% X-view accuracy for the skeleton joints.

### 5.4. Comparison with the State-of-the-Art Models

Finally, we compared our models with the state-of-the-art skeleton-based action recognition models on the NTU-RGBD dataset. In Table 3, we compare our models with those that are based on hand-crafted-features, RNN-based models, and CNN-based models. STGCN outperforms all the models in these categories. This is due to the fact that skeleton data can be better exploited by representing data in a graph structure.

**Figure 9:**
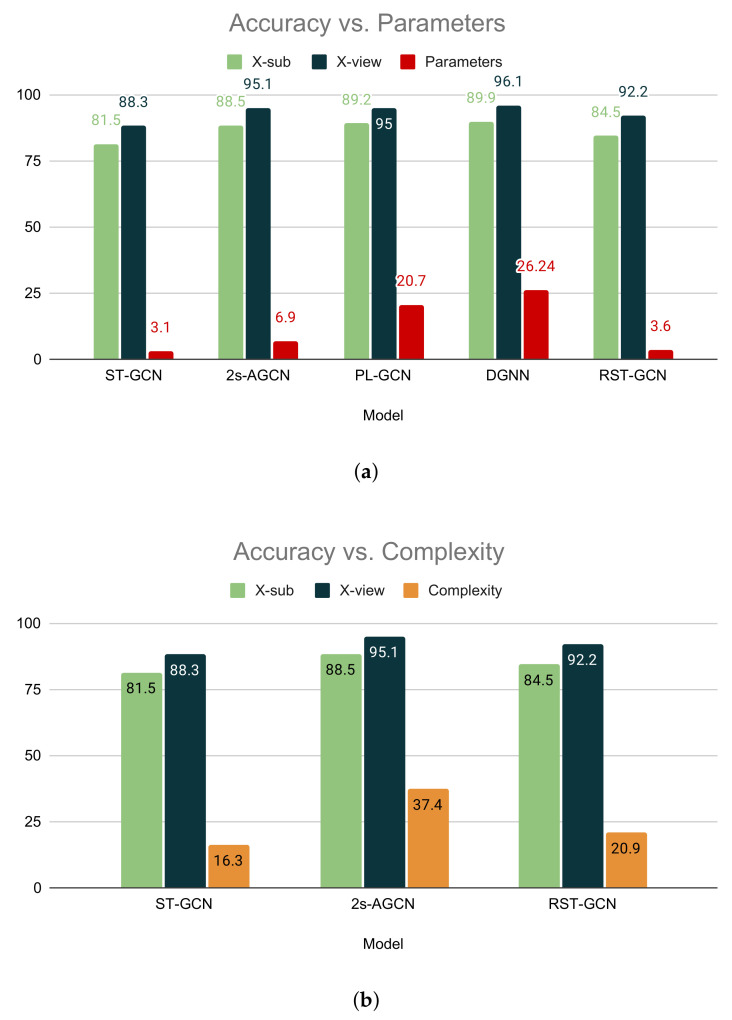
Comparisons between STGCN and other GCN-based models. (**a**) Comparison between STGCN and other GCN-based models with respect to the number of parameters. (**b**) Comparison between STGCN and other GCN-based models with respect to complexity.

The comparison with the GCN-based models is shown in Table 4. Hence, along with the accuracy, we also compare the parameter size (M) and computational complexity in GFLOPs ($10^{9}$ FLOPs). In comparison with ST-GCN [19], our model achieves higher accuracy but is larger than ST-GCN [19] in parameter size. Our model achieves 84.5% and 92.2% accuracy in the X-sub and X-view subsets, respectively, while ST-GCN obtains 81.5% and 88.3% for the same. When we compare our STGCN with 2s-AGCN [20], our model achieves a competitive score in top-1 accuracy, although it is lighter in respect to parameter size and computationally less expensive. Our model has 3.6 M parameters, while 2s-AGCN has 6.9 M parameters. Moreover, our model has a complexity of 20.9 GFLOPs, and 2s-AGCN bears the complexity of 37.4 GFLOPs, which is almost twice the complexity of our model. Although PL-GCN [40] achieves 89.2% X-sub accuracy and 95.0% X-view accuracy, it has a massive size of 20.70 M parameters. Similarly, DGNN [41] has 89.9% X-sub accuracy and 96.1% X-view accuracy with a large parameter size of 26.24 M. In spite of achieving high accuracy, these models are not suitable for deployment in real-life scenarios because of the large parameter size. The comparison between STGCN other GCN-based models in terms of accuracy, parameters, and complexity is illustrated in Figure 9. Our proposed STGCN achieves competitive accuracy with the state-of-the methods because it extracts the temporal and spatial features in parallel, which ensures better feature extraction. In contrast, other methods including ST-GCN, 2s-AGCN, and others extract features sequentially and thus require a large number of parameters. Our model balances the trade-off between accuracy and efficiency, which makes this suitable for deployment in the smart healthcare environment.

### 5.5. Performance Evaluation for Patient Monitoring System

There are nine distinct kinds of activities associated with medical conditions in the NTU-RBGD dataset [13], including *sneeze/cough*, *staggering*, *falling*, *touch head (headache)*, *touch chest (stomachache/heart pain)*, *touch back (backache)*, *touch neck (neckache)*, *nausea or vomiting*, and *use a fan/feeling warm*. The recognition of these activities with high accuracy is of great significance for a real-time patient monitoring system. We evaluated our model on statistical testing methodologies, which are often selected to determine the performance of an HAR classifier. Specifically, we performed a statistical analysis on our model for health-related action categories to evaluate the performance of the model in a real-time patient monitoring system. To measure the effectiveness of our model, we used four metrics, including accuracy, precision, recall, and *F*1-score. These metrics are based on four significant values, which are true positives ($T_{p}$), true negatives ($T_{n}$), false positives ($F_{p}$), and false negatives ($F_{n}$).

Accuracy is defined as the proportion of accurate predictions made across all samples, which is calculated as

(6) $$accuracy=\frac{T_{p}+T_{n}}{T_{p}+F_{p}+T_{n}+F_{n}}.$$

Precision refers to the proportion of correctly predicted results and the total number of observations, which are positively classified. Precision can be defines as

(7) $$precision=\frac{T_{p}}{T_{p}+F_{p}}.$$

Recall is determined as the ratio between correctly predicted results and all the evaluation of the original class. The formula of recall is

(8) $$recall=\frac{T_{p}}{T_{p}+F_{n}}.$$

*F*1-score takes the harmonic mean of precision and recall to create a single score, which is calculated as follows

(9) $$F1-score=\frac{2\times precision\times recall}{precision+recall}.$$

**Figure 10:**
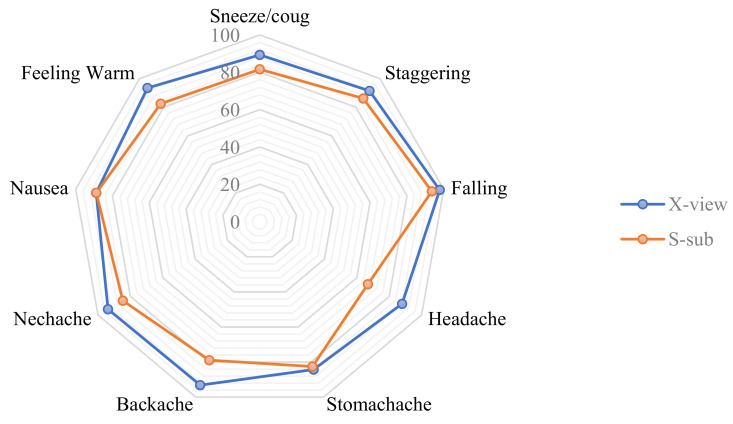
Accuracy of STGCN for medical condition- related actions.

We provide a thorough analysis of our model based on the health-related category in Table 5 and Table 6. As shown in Table 5, on the X-view subset, our model achieves 91% average precision and 92% average recall and *F*1-score. On the X-sub subset, our model is able to achieve 84% average recall, while the average precision and *F*-score are 83%, as shown in Table 6. We also illustrate the accuracy of our STGCN model for these categories in Figure 10. Our proposed model achieves high accuracy in the X-view subset as well as X-sub subset, except for the *touch head (headache)* action. The reason behind achieving a low accuracy in this category in the X-sub subset is that different patients can have pain in different regions of head, and each touches their head differently. However, it is noticeable that our proposed STGCN achieves almost 98% accuracy in the detection of *falling*. In the X-view subset, the model achieves more than 90% accuracy in almost all of the focused categories.

There are a few methods available for patient monitoring systems, while only a few used nine health-related action classes to validate their model. Table 7 compares STGCN with RC VA-LSTM [10] for a patient monitoring system. Out of nine classes, our model outperforms RC VA-LSTM on five categories. However, in four categories, STGCN achieves less accuracy, and the difference is very negligible.

Moreover, we demonstrate the efficiency and applicability of our proposed model in terms of the inference speed in Table 8. We demonstrate the inference speed STGCN with different hardware, including general-purpose CPU (Intel Xeon), high-performance GPU (Nvidia Tesla K80), and an edge device with limited computing resources (Nvidia Jetson Nano). Nvidia Jetson Nano is the most suitable device to perform inference in an actual patient-monitoring. Our model processes 993 frames per second on Nvidia Jetson Nano, which is almost twice as fast as 2s-AGCN [20], and slightly slower than ST-GCN [19]. However, STGCN achieves more than 92.2% accuracy in the X-view subset, which is more than a 4% increase than ST-GCN. Therefore, the proposed model can be used for a wide range of real-time monitoring applications, including patient monitoring.

## 6. Conclusions

We presented a novel architecture for skeleton-based action recognition in patient monitoring and medical condition detection. A spatio-temporal graph convolution operation was used to efficiently learn spatial and temporal features from skeleton data in the proposed STGCN. To make our model suitable for real-world applications, we focused on extracting efficient spatial and temporal features. Our efficient feature extraction method used in STGCN outperformed ST-GCN on NTU-RGBD, a large-scale skeleton-based dataset, with a 4% increase in accuracy while being over 40% more efficient than 2s-AGCN. In addition to consuming less memory, requiring less computation power, and removing the preprocessing overhead, our model can be used for real-time patient monitoring in smart healthcare systems. The tracking of a patient’s condition using data from different sensors along with visual data will be the future direction of this research.

## Figures and Tables

**Table 1:** Comparison of the validation with the X-view accuracy of STGCN with or without different parameters.

Methods	Accuracy
STGCN (w/o A)	91.8
STGCN (w/o B)	91.6
STGCN (w/o C)	91.8
STGCN	92.2

**Table 2:** Evaluation of STGCN on different input types.

Input Features	X-Sub (%)	X-View (%)
Bone data	83.8	91.4
Joint data	84.5	92.2

**Table 3:** Comparisons between STGCN and other state-of-the-art methods on the NTU-RGBD dataset.

Methods	X-Sub (%)	X-View (%)
Lie Group [26]	50.1	82.8
Deep LSTM [13]	60.7	67.3
ST-LSTM [36]	69.2	77.7
STA-LSTM [37]	73.4	81.2
VA-LSTM [27]	79.2	87.7
ARRN-LSTM [38]	81.8	89.6
Ind-RNN [28]	81.8	88.0
Two-Stream 3DCNN [30]	66.8	72.6
TCN [15]	74.3	83.1
Clips + CNN + MTLN [31]	79.6	84.8
Synthesized CNN [16]	80.0	87.2
CNN + Motion + Trans [39]	83.2	89.3
RSR-GCN (ours)	84.5	92.2

**Table 4:** Comparisons of STGCN with state-of-the-art GCN-based methods on the NTU-RGBD dataset.

Methods	X-Sub (%)	X-View (%)	Parameters (M)	Complexity (GFLOPS)
ST-GCN [19]	81.5	88.3	3.1	16.3
2s-AGCN [20]	88.5	95.1	6.9	37.4
PL-GCN [40]	89.2	95.0	20.7	-
DGNN [41]	89.9	96.1	26.24	-
STGCN (ours)	84.5	92.2	3.6	20.9

**Table 5:** Classification report of STGCN of healthcare-related actions in the X-view subset.

Action	Precision	Recall	F1-Score	Accuracy (%)
Sneeze/cough	0.89	0.88	0.88	89.35
Staggering	0.91	0.95	0.93	91.44
Falling	0.98	0.99	0.99	97.82
Headache	0.88	0.93	0.90	87.99
Stomachache/heart pain	0.84	0.90	0.87	84.27
Backache	0.93	0.90	0.92	93.14
Neck ache	0.94	0.91	0.92	93.77
Nausea/vomiting	0.88	0.90	0.89	88.44
Feeling warm	0.93	0.95	0.94	93.46

**Table 6:** Classification report of STGCN of healthcare-related actions in the X-sub subset.

Action	Precision	Recall	F1-Score	Accuracy (%)
Sneeze/cough	0.82	0.63	0.71	81.60
Staggering	0.86	0.97	0.91	86.17
Falling	0.94	0.95	0.94	93.55
Headache	0.67	0.76	0.71	66.88
Stomachache/heart pain	0.83	0.87	0.85	82.53
Backache	0.79	0.87	0.83	78.95
Neck ache	0.85	0.78	0.81	84.58
Nausea/vomiting	0.89	0.87	0.88	88.81
Feeling warm	0.82	0.89	0.86	82.49

**Table 7:** Comparison of accuracy of medical condition-related actions.

Action	RC VA-LSTM Accuracy (%)	STGCN Accuracy (%)
Sneeze/cough	86.30	89.35
Staggering	96.00	91.44
Falling	98.70	97.82
Headache	81.30	87.99
Stomachache/heart pain	85.30	84.27
Backache	86.10	93.14
Neck ache	84.30	93.77
Nausea/vomiting	90.40	88.44
Feeling warm	91.40	93.46

**Table 8:** Comparisons of STGCN with state-of-the-art GCN-based methods in terms of inference speed.

Methods	Inference Speed (FPS)		
	CPU	Jetson Nano	Nvidia K80
ST-GCN [19]	273	1037	5733
2s-AGCN [20]	132	528	2948
Proposed	248	993	5539

## Data Availability

Not applicable.
